# Novel Model of Oxalate Diet-Induced Chronic Kidney Disease in Dahl-Salt-Sensitive Rats

**DOI:** 10.3390/ijms241210062

**Published:** 2023-06-13

**Authors:** Prabhatchandra Dube, Vaishnavi Aradhyula, Apurva Lad, Fatimah K. Khalaf, Joshua D. Breidenbach, Eshita Kashaboina, Snigdha Gorthi, Shangari Varatharajan, Travis W. Stevens, Jacob A. Connolly, Sophia M. Soehnlen, Ambika Sood, Amulya Marellapudi, Meghana Ranabothu, Andrew L. Kleinhenz, Oliver Domenig, Lance D. Dworkin, Deepak Malhotra, Steven T. Haller, David J. Kennedy

**Affiliations:** 1 Department of Medicine, University of Toledo College of Medicine and Life Sciences, Toledo, OH 43606, USA; vaishnavi.aradhyula@rockets.utoledo.edu (V.A.);; 2 Department of Medicine, University of Alkafeel College of Medicine, Najaf 54001, Iraq; 3 Attoquant Diagnostics GmBH, 1110 Vienna, Austria

**Keywords:** chronic kidney disease, uremic cardiomyopathy, animal model

## Abstract

Diet-induced models of chronic kidney disease (CKD) offer several advantages, including clinical relevance and animal welfare, compared with surgical models. Oxalate is a plant-based, terminal toxic metabolite that is eliminated by the kidneys through glomerular filtration and tubular secretion. An increased load of dietary oxalate leads to supersaturation, calcium oxalate crystal formation, renal tubular obstruction, and eventually CKD. Dahl-Salt-Sensitive (SS) rats are a common strain used to study hypertensive renal disease; however, the characterization of other diet-induced models on this background would allow for comparative studies of CKD within the same strain. In the present study, we hypothesized that SS rats on a low-salt, oxalate rich diet would have increased renal injury and serve as novel, clinically relevant and reproducible CKD rat models. Ten-week-old male SS rats were fed either 0.2% salt normal chow (SS-NC) or a 0.2% salt diet containing 0.67% sodium oxalate (SS-OX) for five weeks.Real-time PCR demonstrated an increased expression of inflammatory marker interleukin-6 (IL-6) (*p* < 0.0001) and fibrotic marker Timp-1 metalloproteinase (*p* < 0.0001) in the renal cortex of SS-OX rat kidneys compared with SS-NC. The immunohistochemistry of kidney tissue demonstrated an increase in CD-68 levels, a marker of macrophage infiltration in SS-OX rats (*p* < 0.001). In addition, SS-OX rats displayed increased 24 h urinary protein excretion (UPE) (*p* < 0.01) as well as significant elevations in plasma Cystatin C (*p* < 0.01). Furthermore, the oxalate diet induced hypertension (*p* < 0.05). A renin–angiotensin–aldosterone system (RAAS) profiling (via liquid chromatography–mass spectrometry; LC–MS) in the SS-OX plasma showed significant (*p* < 0.05) increases in multiple RAAS metabolites including angiotensin (1–5), angiotensin (1–7), and aldosterone. The oxalate diet induces significant renal inflammation, fibrosis, and renal dysfunction as well as RAAS activation and hypertension in SS rats compared with a normal chow diet. This study introduces a novel diet-induced model to study hypertension and CKD that is more clinically translatable and reproducible than the currently available models.

## 1. Introduction

Chronic Kidney Disease (CKD) is an established and recognized leading public health concern. According to the Centers for Disease Control and Prevention (CDC), one in seven adults in the United States have CKD. CKD is defined by decreasing kidney function or a declining glomerular filtration rate (GFR) indicated by several stages. An adult patient is identified with CKD when they present with a GFR lower than 60 mL/min/1.73 m^2^, with evidence of renal injury, histological changes on kidney biopsy, electrolyte abnormalities, albuminuria, creatinemia, and hypertension for three or more months [1,2]. Diabetes, hypertension, and dyslipidemia are risk factors and can propagate glomerular and tubular injury via inflammation [3]. The subsequent repair and recovery of tissue function initiates the development of fibrosis. This eventually leads to irreversible kidney failure, or end-stage renal disease (ESRD), leading to extremely high morbidity and mortality in these patients. CKD patients also exhibit an elevated cardiovascular disease (CVD) risk, manifesting as coronary artery disease, heart failure, arrhythmias, and sudden cardiac death [4,5]. Rather than ESRD itself, CVD is the leading cause of death, accounting for 40% to 50% of all deaths in advanced CKD patients [6]. 

Hypertension is one of the leading causes of CKD and has been reported to occur in 85% to 95% of patients with CKD in later stages [7,8]. Long-term uncontrolled hypertension causes high interglomerular pressure, impairs glomerular filtration, and leads to microalbuminuria or proteinuria [9]. Dahl salt-sensitive (SS) rats are an established inbred, diet-induced model of hypertension, renal damage, and immune cell infiltration that are widely used to study salt-sensitive hypertension. SS rats also develop hypertensive CKD at approximately 10 weeks with increased serum creatinine, urinary protein excretion (UPE), enlarged glomerular size, and severe tubulointerstitial fibrosis.

Mulay et al. has previously demonstrated how a calcium-free oxalate diet can induce acute kidney injury, CKD, and renal failure via tubular injury [10]. Oxalate imbalance can lead to hyperoxaluria, which predisposes urinary calcium oxalate supersaturation, the formation of crystals consistent with nephrolithiasis, and apoptotic and inflammatory responses consistent with acute kidney injury. Eventual tubulointerstitial injury activates the renin–angiotensin–aldosterone-system (RAAS) [10,11]. 

The present study aims to contrast the oxalate diet-induced models and characterize the etiology of CKD by establishing an oxalate and hypertension-induced model. Our objective is to determine whether the addition or absence of an oxalate diet could exacerbate hypertension-induced CKD. We used SS rats for five reasons: (1) 30% to 50% of all adults with hypertension exhibit SS hypertension [7]; (2) it is well established that SS rats experience features of CKD such as cardiac hypertrophy and fibrosis [12,13], medullary protein cast formation, renal interstitial fibrosis, and inflammation [14]; (3) the normotensive control Dahl salt-resistant inbred strain displays only a minor increase in blood pressure when placed on a high-salt diet, demonstrating a genetically divergent strain of salt sensitivity; (4) using rats of the same strain allows for a decrease in genetic variability and confounding variables, a limitation in the previous study on oxalate-induced hypertension [10]; and (5) it is critical to develop a model establishing a genetically divergent SS strain selective for hypertension-induced CKD that is more characteristic of the disease pathophysiology. We hypothesized that the addition of dietary oxalate to the SS model would produce a clinically relevant model for CKD. 

## 2. Results

### 2.1. Oxalate Diet Demonstrates Blood Chemistry Abnormalities Indicating Renal Dysfunction

**Figure 1:**
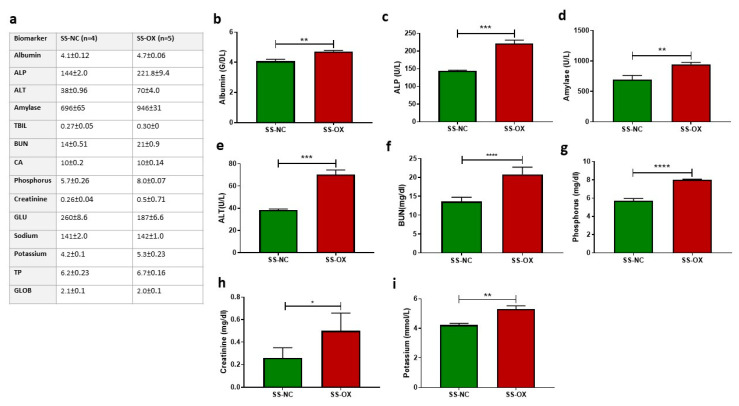
Blood biochemistry analysis (**a**) of Dahl-Salt-Sensitive rats on oxalate diet (SS-OX) display increased serum albumin (*p* = 0.0019) (**b**), increased serum alkaline phosphate (ALP) (*p* = 0.0002) (**c**), increased serum amylase (*p* = 0.0075) (**d**), increased serum alanine transaminase (ALT) (*p* = 0.0002) (**e**), increased blood urea nitrogen (BUN) (*p* < 0.0001) (**f**), increased serum phosphorus (*p* < 0.0001) (**g**), increased serum creatinine *p* = 0.0183 (**h**), and increased serum potassium (*p* = 0.0057) (**i**), compared with Dahl-Salt-Sensitive rats on a normal chow diet (SS-NC) (*t*-test, * *p* < 0.05, ** *p* < 0.01, *** *p* < 0.001, **** *p* < 0.0001, *n* = 4–5).

To investigate renal function, we obtained the comprehensive metabolic panels from the SS-OX and SS-NC rat groups (Figure 1a). The SS-OX rats presented with increased serum albumin (Figure 1b), ALP (Figure 1c), amylase (Figure 1d), ALT (Figure 1e), BUN (Figure 1f), phosphorous (Figure 1g), creatinine (Figure 1h), and potassium (Figure 1i) compared with the SS-NC rats, consistent with significant renal dysfunction. These changes can be attributed to the increased tubulointerstitial oxalate crystal deposition in the SS-OX compared to SS-NC rats (Figure S1). 

### 2.2. Oxalate Diet Induces Increased Renal Inflammation 

**Figure 2:**
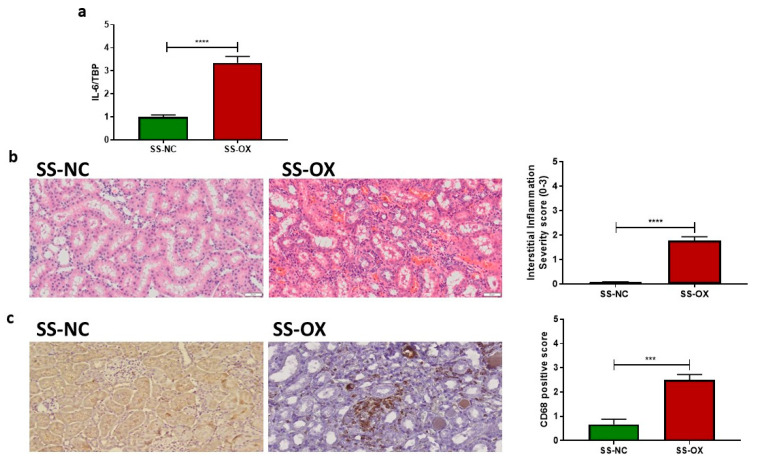
Dahl-Salt-Sensitive rats on an oxalate diet (SS-OX) displayed a marked increase in renal inflammation, pro-fibrotic factors, and immune cell proliferation compared with Dahl-Salt-Sensitive rats on a normal chow diet (SS-NC). SS-OX presented greater levels of pro-inflammatory cytokine IL-6 (*p* < 0.0001) (**a**), interstitial inflammation (*p* < 0.0001) (**b**), and levels of CD-68 indicating increased macrophage infiltration (*p* = 0.0001) (**c**) vs. SS-NC (Magnification: 50 µM, *t*-test, *** *p* < 0.001, **** *p* < 0.0001, *n* = 10–16).

Quantitative real-time PCR was performed on both the SS-OX and SS-NC kidney samples to establish the molecular evidence of renal inflammation by examining the expression of Interleukin-6 (IL-6). We observed a significant upregulation in the expression of IL-6 in the SS-OX compared with the SS-NC samples (Figure 2a). SS-OX kidneys also displayed greater interstitial inflammation compared with SS-NC kidneys, noted by increased macrophage infiltration (Figure 2b). Furthermore, immunohistochemical analysis on kidney tissues from the SS-OX rats demonstrated an increase in levels of CD-68 (Figure 2c), which is consistent with the increased inflammatory response shown in the real-time PCR and H&E staining (Figure 2c).

### 2.3. Oxalate Diet Elicits Increased Renal Fibrosis and Injury 

**Figure 3:**
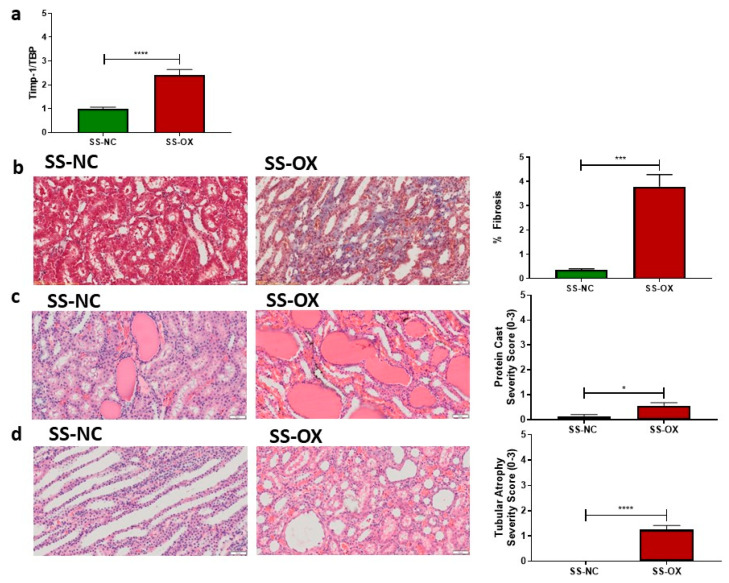
Dahl-Salt-Sensitive rats on an oxalate diet (SS-OX) displayed an increased presence of fibrosis-related gene tissue inhibitors of metalloproteinase (TIMP-1) (*p* < 0.0001) (**a**), increased renal fibrosis (*p* = 0.0008) (**b**), and kidney injury indicated by increased protein casts (*p* = 0.0239) (**c**) and tubular atrophy (*p* < 0.0001) (**d**) compared with Dahl-Salt-Sensitive rats on a normal chow diet (SS-NC) (Magnification: 50 µM, *t*-test, * *p* < 0.05, *** *p* < 0.001, **** *p* < 0.0001, *n* = 10–16).

Compared with SS-NC, the SS-OX kidney samples demonstrated an upregulated expression of the fibrosis-related gene tissue inhibitor of metalloproteinase (TIMP-1) (Figure 3a). Consistent with these findings, on trichrome staining, SS-OX kidneys displayed increased renal fibrosis compared with SS-NC kidneys (Figure 3b). Histological analyses of the kidney samples on H&E staining displayed increased protein casts (Figure 3c) and a greater extent of tubular atrophy, indicating kidney injury (Figure 3d) in SS-OX when compared with SS-NC.

### 2.4. Oxalate Diet Activates RAAS and Induces Renal Dysfunction

**Figure 4:**
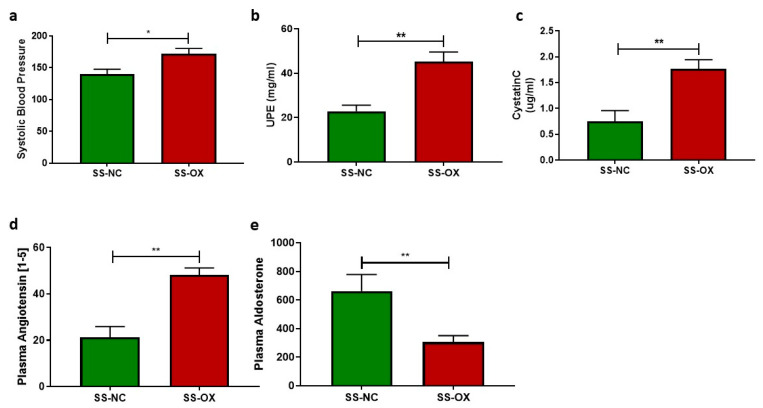
Dahl-Salt-Sensitive rats on an oxalate diet (SS-OX) displayed increased hypertension, glomerular filtration rate, and renin angiotensin aldosterone system (RAAS) activation when compared with Dahl-Salt-Sensitive rats on a normal chow diet (SS-NC) (**a**) and increased urine protein excretion (UPE) (*p* = 0.0146) (**b**), increased levels of levels of cystatin C (*p* = 0.0064) (**c**), increased plasma angiotensin (*p* = 0.0015) (**d**), and decreased serum aldosterone (*p* = 0.0068) (**e**) (*t*-test, * *p* < 0.05, ** *p* < 0.01, *n* = 3–6).

The SS-OX and SS-NC demonstrated hypertension via high BP, but the SS-OX models displayed a greater increase in systolic arterial BP taken by a non-invasive tail-cuff compared with the SS-NC (Figure 4a). The SS-OX rats also demonstrated a statistically significant increase in concentrations of UPE compared with SS-NC (Figure 4b). Similarly, SS-OX rats showed increased levels of Cystatin C compared with SS-NC, indicating renal dysfunction (Figure 4c). The oxalate diet also affected RAAS metabolism, shown by increased plasma angiotensin and suppressed levels of aldosterone, as seen in the SS-OX rats (Figure 4d,e).

### 2.5. Oxalate Diet Induces Cardiac Inflammation and Fibrosis

**Figure 5:**
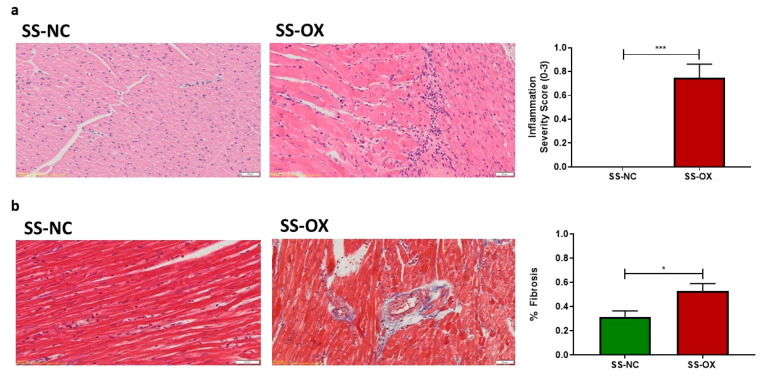
Cardiac fibrosis and inflammation were markedly elevated in Dahl-Salt-Sensitive rats on an oxalate diet (SS-OX) when compared with Dahl-Salt-Sensitive rats on a normal chow diet (SS-NC). SS-OX rats displayed greater interstitial cardiac inflammation (*p* = 0.0001, *n* = 8–16) (**a**) and increased perivascular cardiac fibrosis (*p* = 0.0466, *n* = 4–8) (**b**) (Magnification: 50 µM, *t*-test, * *p* < 0.05, *** *p* < 0.001).

To examine cardiac injury, an H&E stain on cardiac tissue slides was performed and revealed increased interstitial inflammation in SS-OX tissues compared with the SS-NC (Figure 5a). Furthermore, trichrome staining on cardiac tissue slides demonstrated increased fibrosis in the SS-OX rats compared with the SS-NC rats (Figure 5b).

### 2.6. Oxalate Diet Induces Pathological Cardiac Hypertrophy and Left Ventricular Dysfunction

**Figure 6:**
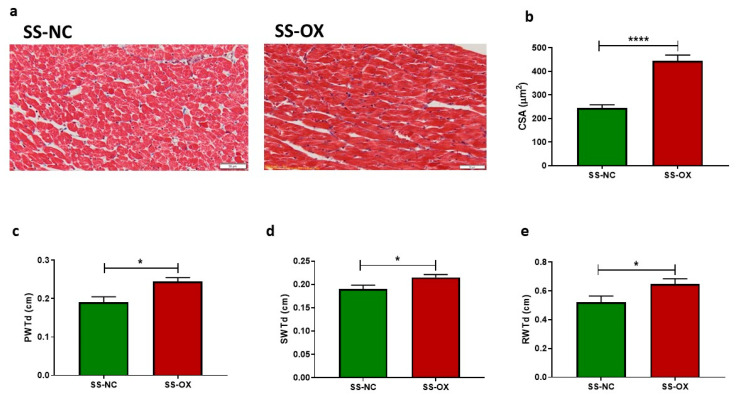
Dahl-Salt-Sensitive rats on an oxalate diet (SS-OX) presented pathological cardiac hypertrophy and left ventricular dysfunction, indicating uremic cardiomyopathy, when compared with Dahl-Salt-Sensitive rats on a normal chow diet (SS-NC). SS-OX rats displayed cardiac hypertrophy via increased cross-sectional area (CSA) of cardiomyocytes compared with SS-NC rats (*p* < 0.0001) (**a**,**b**). SS-OX rats also displayed increased heart posterior wall thickness (PWTd) (*p* = 0.0111) (**c**), increased septal wall thickness (SWTd) (*p* = 0.0452) (**d**), and increased relative wall thickness (RWTd) (*p* = 0.0423) (**e**) compared with SS-NC models (Magnification: 50 µM, *t*-test, * *p* < 0.05, **** *p* < 0.0001, *n* = 6–8).

To further understand the changes in cardiac remodeling indicative of uremic cardiomyopathy, cardiac tissue was trichrome stained. SS-OX cardiac tissue demonstrated increased cardiac hypertrophy through increased CSA compared with SS-NC tissue (Figure 6a,b). In addition, SS-OX cardiac tissue also demonstrated increased heart PWTd (Figure 6c), increased SWTd (Figure 6d), and increased RWTd (Figure 6e) compared with the SS-NC models.

## 3. Discussion

Several animal models have been developed to study CKD pathogenesis, but these models do not precisely simulate human disease due to several limitations and weaknesses. In vitro models do not study cell-to-cell signaling pathways and the in vivo responses of disease progression. Genetic models offer the opportunity to study the genetic etiology of CKD, but do not display the mechanisms of glomerular disease. The most widely used rat model is one of unilateral ureteral obstruction (UUO). This model demonstrates significant damage to renal function by preventing urine output; however, it excludes biomarker discovery, an essential factor in monitoring CKD complications [15,16]. The 5/6 nephrectomy model (also known as the Renal Mass Reduction model) is also widely used for studying CKD, but the need for surgical intervention is a strong limitation that causes variability in the model’s reproducibility [17]. While this model has been shown to recapitulate key features of uremic cardiomyopathy in both the rat [18,19,20,21] and mouse [22] including proteinuria, hypertension, diastolic dysfunction, left ventricular hypertrophy, and fibrosis, this model has also demonstrated increased albuminuria without increased blood pressure or cardiac fibrosis [23]. Therefore, the research of CKD is limited by a lack of holistic, inducible, reproducible, and translational experimental models. 

Given that intrinsic susceptibility to hypertension is already present in the SS rat model, the addition and absence of an extrinsic dietary component such as oxalate could stimulate a clinically relevant and novel CKD animal model. In the present study, we observed that the SS-OX model demonstrated several key aspects of the prognosis of CKD via blood biomarkers, such as an increased presence of ALP, amylase, ALT, phosphorous, BUN, creatinine, and potassium compared with the SS-NC. This is consistent with previous research associating oxalate diet-induced injury to CKD pathology [10]. ALP is a prognostic marker for CKD [24] that modulates the balance between bone mineralization and cardiovascular calcification. Increased levels of ALP pathologically correlate with increased inflammation, vascular calcification, and endothelial dysfunction. Increased serum amylase is a standard diagnostic tool for recognizing pancreatitis and has also been shown to be elevated in CKD and ESRD due to impaired renal clearance. ALT, a marker of liver injury, has been shown to be significantly higher in CKD patients than non-CKD patients [25]. As expected, SS-OX rats also present increased serum phosphorous levels due to impaired clearance from a weakened glomerular filtration barrier. An oxalate diet also induced a decline in GFR and an increase in BUN and creatinine, the most significant markers in identifying kidney dysfunction. Finally, hyperkalemia seen in SS-OX rats is correspondent with renal dysfunction, resulting in decreased potassium excretion [16]. Interestingly, the pattern of increased ALP, ALT, and amylase also suggests hepatic and pancreatic injury. This is similar to cases of primary hyperoxaluria type 1, a rare autosomal, recessive, inherited metabolic disease in which the systemic deposition of calcium oxalate crystals leads to early kidney failure and triggers acute liver injury [26]. Pancreatic injury, on the other hand, appears to occur secondarily to oxalate-induced nephropathy, rather than concurrently [27]. 

Another critical component in the pathogenesis of CKD is the inflammation and infiltration of pro-inflammatory cytokines, as seen in the SS-OX compared with the SS-NC model. The calcium oxalate deposits in the renal tubules are associated with increased renal cell injury, cell loss, inflammation, and fibrosis [28,29]. Exposure to these crystals induces the release of fibrotic factors and activates endothelin-1, which generates pro-inflammatory cytokines and the upregulation of IL-6 [30]. IL-6 is a major recruitment factor for monocytes and macrophages. CD-68 is the most reliable marker for macrophages [30]. Therefore, IL-6 and CD-68 are both significant markers of macrophage proliferation. Additionally, CD-68 levels have been associated with the progression of CKD and renal fibrosis via glomerular disease [30,31]. Endothelin-1 also triggers pro-fibrotic markers, such as Timp-1, which attempt to reconstruct and restabilize the glomerular basement membrane for structural integrity. However, when these repair mechanisms are disrupted, such as in the overactivation of the RAAS system [32], the process can present as fibrosis. Tubulointerstitial fibrosis is a component of CKD and has been shown to be the best predictor of progression toward ESRD [33]. In the present study, lymphocyte infiltration, proliferation, and fibrotic tissue were evident in the SS-OX rats. However, inflammation and fibrosis were not evident in the SS-NC rats in the absence of 5 weeks of an oxalate diet, although both the SS-OX and SS-NC are equally susceptible to hypertension-induced CKD. This suggests that the SS-OX rats had sustained more significant tubulointerstitial renal injury in a shorter timeframe, likely due to the addition of the oxalate diet. Additionally, SS rats have been previously studied to demonstrate renal interstitial fibrosis and inflammation in the form of T-cell and macrophage infiltrates; however, significant fibrosis was only visible when the diets were altered from 0.4% low salt diet to 4.0% high salt diet at 9 weeks [34]. 

Other histological changes observed include increased protein casts and tubular atrophy in the SS-OX compared with the SS-NC rats. Hyaline casts are formed from mucoprotein secreted by renal tubular cells and are indicative of renal tubular injury [35]. Tubular atrophy is also associated with chronic tubular injury that is a consequence of decreased glomerular filtration [36,37], and, along with interstitial fibrosis, has been recognized as the most predictive marker of GFR decline [38]. Protein casts and tubular injury could be a result of compromised blood flow to the kidney, resulting in kidney injury comparable to CKD. SS-OX rats demonstrated increased protein cast formation and excretion. Increased UPE, likely from tubulointerstitial damage and decreased reabsorption, is considered the most significant risk factor for the progression of CKD [36]. Serum creatinine was also elevated in the SS-OX rats compared with the SS-NC rats, indicating a decrease in GFR. GFR is critical in the diagnosis and staging of CKD [39], and low GFR is a potent predictor of CVD and early mortality [40]. Creatinine is generally used as the biomarker for filtration; however, since it can be affected by diet, muscle mass, rapidly changing kidney function, and active secretion by the kidney, cystatin C was also measured. Cystatin C can better predict adverse cardiovascular outcomes and has been shown to be unaffected in certain inflammatory and metabolic conditions [32,41]. 

Both SS-OX and SS-NC rats are hypertensive, but the pathophysiology of the hypertension is distinguishable between the two models. The SS-OX rats displayed an increase in plasma angiotensin and a decrease in plasma aldosterone. This was significantly more affected in the SS-OX compared with the SS-NC models, indicating that the oxalate diet-induced renal damage had triggered an enhancement of the RAAS system. Angiotensin II is known to be the primary metabolite that is activated by the kidney when renal blood flow is compromised. Angiotensin II is also a renal growth factor, and can induce renal hyperplasia and hypertrophy, activate tubulointerstitial fibrosis, and increase the expression of pro-inflammatory and pro-fibrotic factors [42]. This effect can explain the increased fibrosis and inflammation seen in SS-OX tissues. Additionally, aldosterone levels were decreased in the SS-OX rats, as the suppression of aldosterone via a negative feedback mechanism from the elevated potassium levels suggests a protective mechanism in CKD [42]. This has also been observed in human models [42].

In the present study, the SS-OX rats demonstrated an increased cardiac fibrosis in the epicardial vessels, and an increased cardiac interstitial inflammation compared with the SS-NC rats. The SS-OX rats also displayed cardiac hypertrophy indicated by increased cardiomyocyte CSA, and increased posterior, septal, and relative wall thickness. Coronary microvascular dysfunction provides a plausible mechanism for CKD-induced myocardial damage leading to the phenomenon of uremic cardiomyopathy [43,44]. Left ventricular hypertrophy (LVH) is often present in ESRD due to the activation of the RAAS system, hypertension, arterial stiffness, and hyperphosphatemia [44]. In fact, epidemiological studies using magnetic resonance imaging suggest that the primary manifestation of uremic cardiomyopathy is LVH [45]. Increased cardiac fibrosis due to diffuse collagen deposition between capillaries and cardiomyocytes further exacerbates maladaptive ventricular hypertrophy [43,46]. In addition, vascular smooth muscle cells can calcify due to hypertensive and hemodynamic changes from the RAAS activation observed in CKD [47]. The subsequent increase in cardiac afterload and heart failure induces LVH, furthering the process of uremic cardiomyopathy [48,49,50]. Fibrosis in early CKD results in the calcification of epicardial vessels. Vascular calcification is described as a more acute process in the early stages of CKD [51], as seen in the SS-OX models in this study. This contrasts with valvular calcifications, which are seen in later stages of CKD, or ESRD [52]. Additionally, albuminuria, seen in the present study via increased UPE in the SS-OX rats, has been identified as an indicator of cardiorenal risk in the renal and nonrenal populations [53,54]. Higher levels of albuminuria indicate a graded increase in mortality risk independent of GFR [53]. The SS-OX model provides a clinically relevant diet-induced model of hypertension-induced uremic cardiomyopathy by reliably inducing CKD complications such as significant RAAS activation, hypertension, cardiac fibrosis, inflammation, LVH, and cardiac remodeling [43]. 

## 4. Materials and Methods

### 4.1. Animal Models for Oxalate Diet-Induced CKD 

**Figure 7:**
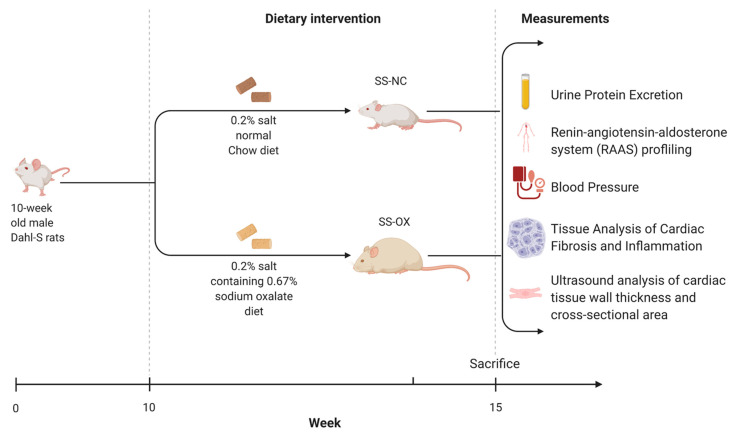
This graphic displays the methods and timeframe used in this study with Dahl-Salt Sensitive (SS) rats fed a normal chow diet (SS-NC) and fed an oxalate diet (SS-OX) to develop the oxalate diet-induced CKD model. The measurements taken after the rats were euthanized 15 weeks later include urine protein excretion, renin–angiotensin–aldosterone-system (RAAS) profiling, blood pressure measurements, kidney collection to assess histological changes in renal architecture, and ultrasound analysis to identify cardiac remodeling.

All animal studies were performed in accordance with the National Institutes of Health’s Guide for the Care and Use of Laboratory Animals and were approved by the Institutional Animal Care and Use Committee at the University of Toledo. SS rats were obtained from Charles River (SSMcwi, hereafter called SS rats). Age-matched, ten-week-old male rats were maintained on either a 0.2% salt normal chow (SS-NC) or a 0.2% salt diet containing 0.67% sodium oxalate (SS-OX) for five weeks, as previously shown to stimulate oxalate-induced CKD [10] (Figure 7). The SS-OX model and the SS-NC were administered equal amounts of sodium concentration in their diets. The oxalate diet was prepared by adding 50 umol/g sodium oxalate to a calcium-free standard diet (Ssniff, Soest, Germany). The rats were fed ad libitum with unlimited access to food and water, and all were kept at a 12 h light cycle. After blood was collected and blood pressure measurements were retrieved (described below), the rats were euthanized at 15 weeks of age via thoracotomy and exsanguination via cardiac puncture while anesthetized under isoflurane anesthesia (5% in 100% O_2_ administered via nose cone). 

### 4.2. Blood Biochemistry Analysis

Whole blood was collected from saphenous vein and 200 µL was loaded onto Abaxis rotor with VetScan2 Chemistry Analyzer (Ref: 500-0038, Abaxis, Union City, CA, USA). A comprehensive diagnostic profile comprising alanine aminotransferase (ALT), albumin (ALB), alkaline phosphatase (ALP), amylase, globulin (GLOB), glucose (GLU), blood urea nitrogen (BUN), total protein (TP), calcium, sodium, potassium, and phosphorus was conducted.

### 4.3. Reverse Transcription-Quantitative Polymerase Chain Reaction (RT-qPCR) and RNA Isolation

RNA extraction, cDNA preparation, and RT-qPCR were all performed utilizing the QIAGEN (Germantown, MD, USA) automated workflow system which utilizes the QIAgility and QIAcube HT liquid handling robots. RNA from the kidney tissue was isolated utilizing the QIAzol/Chloroform extraction methodology via automated liquid handling equipment (QIAcube HT). Approximately 500 ng of extracted RNA was used to synthesize cDNA (QIAGEN’s RT2 First Strand Kit cat #330404). RT-PCR was then performed utilizing QIAGEN’s Rotor-Gene Q thermocycler. The calculation of gene expression was conducted by comparing the relative change in cycle threshold value (ΔCt). Fold change in expression was also calculated using the 2-ΔΔCt equation as previously described [55].

### 4.4. Renal and Cardiac Histology

Kidneys and hearts from the SS-NC and SS-OX rats were fixed in 4% formaldehyde (pH 7.2), paraffin embedded, and cut into 4 μm sections. These tissue sections were deparaffinized with xylene and rehydrated by sequential incubations in ethanol and water. CD-68 antibodies were purchased from Abcam (Cambridge, MA, USA). A Vectastain Elite-ABC kit (Vector Labs) (Burlingame, CA, USA) was used following the manufacturer’s protocol. Trichrome and hematoxylin and eosin (H&E) staining was then conducted on the 4 μm renal tissue and cardiac tissue sections. For each 4 μm section, 10 images were randomly taken with a bright-field microscope with 20× lens. Quantitative morphometric analysis was performed using automated and customized algorithms/scripts for batch analysis (ImageIQ Inc., Cleveland, OH, USA) written for Image Pro Plus 7.0, as our team has previously described in detail [55,56,57,58]. Renal and cardiac histology was then graded in a blinded fashion and scored on a scale of 0 to 4 for inflammation, fibrosis, and injury. 

### 4.5. Blood Pressure Measurements

Systolic arterial blood pressure (BP) was measured using a volume-pressure recording tail-cuff method (CODA non-invasive BP system, Kent Scientific Corporation, Torrington, CO, USA) [59,60]. Conscious rats were allowed to acclimatize for 15–20 min. All animal temperatures were maintained between 30 and 35 °C before acquiring blood pressure readings. Ten acclimation cycles, followed by a minimum of 20 measurement cycles, were recorded. Any movement or sign of stress during measurement was noted and measurement was excluded accordingly. BP was also assessed by radio telemetry for 14 h following 5 weeks of oxalate diet. The values of BP obtained by the two methods were highly correlated. 

### 4.6. Angiotensin Metabolite Profiling with Mass Spectrometry (RAAS-Fingerprinting)

Equilibrium concentrations of angiotensin peptides and aldosterone were quantified in plasma samples by liquid chromatography–mass spectrometry/mass spectroscopy (LC–MS) performed at a commercial laboratory (Attoquant Diagnostics, Vienna, Austria), using previously validated and described methods [61,62,63]. Briefly, samples were spiked with a stable isotope-labeled internal standard for each angiotensin and a deuterated internal standard for aldosterone (aldosterone D4) after ex vivo equilibration, and analytes were extracted using C18-based solid-phase extraction. Extracted samples were analyzed using LC–MS analysis with a reversed-analytical column (Acquity UPLC C18, Waters), operating in line with a XEVO TQ-S triple quadrupole mass spectrometer (Waters Xevo TQ/S, Milford, MA, USA) in a multiple reaction monitoring mode. Internal standards were used to correct for analyte recovery across the sample preparation procedure in each individual sample. Analyte concentrations were calculated from integrated chromatograms, considering the corresponding response factors determined in appropriate calibration curves in serum matrix, when integrated signals exceeded a signal-to-noise ratio of 10.

### 4.7. Echocardiography 

Left ventricular functions of SS-rats were evaluated by echocardiography, assessing for heart posterior wall thickness (PWTd), septal wall thickness (SWTd), and relative wall thickness (RWTd), as we have previously described [56]. After hearts were extracted and cardiac tissue was processed, a cardiomyocyte cross-sectional area (CSA) was quantified. 

### 4.8. Measurement of Cystatin C and Urine Protein Excretion Rate

Cystatin C and Urine Protein Excretion (UPE) in 24 h urine samples were measured by ELISA purchased from Biovision (Milpitas, CA, USA) and performed in accordance with the manufacturers’ protocol.

### 4.9. Statistical Analysis

Data are presented as the mean ± standard of error. The Student’s Unpaired *t*-test was used to assess statistically significant differences between two groups. One-way ANOVA and post hoc multiple comparisons tests were used when comparing more than two groups. The study was powered based on detecting a 30% increase in the primary outcome plasma cystatin C levels. In order to achieve 85% power, using a 2-tailed test, with an overall alpha = 0.05 and beta = 0.15, we calculated a sample size of 8 animals per group. All statistical analysis was performed using GraphPad Prism 8.0 software. Statistical significance was accepted as *p* < 0.05.

## 5. Conclusions

The addition of oxalate to salt-sensitive, hypertensive rats induced moderate to advanced CKD in a short time frame of 5 weeks, while generating more profound hypertension compared with the absence of an oxalate diet. The tubulointerstitial deposition of oxalate crystals produced renal injury, inflammation, renal and cardiac fibrosis, cardiac remodeling, and uremic cardiomyopathy. This study establishes a novel, foundational animal model that is translational, reproducible, and characteristic of the cardiovascular implications of CKD. 

## Data Availability

The datasets generated or analyzed during the current study are available from the corresponding author on reasonable request.

## Supplementary Materials

The following supporting information can be downloaded at: https://www.mdpi.com/article/10.3390/ijms241210062/s1.
