# Supplementary material for: Novel Model of Oxalate Diet-Induced Chronic Kidney Disease in Dahl-Salt-Sensitive Rats
Source: Int J Mol Sci. 2023 Jun 13;24(12):10062. doi: 10.3390/ijms241210062 (PMC10298248; doi:10.3390/ijms241210062)
Supplement: Supplementary file 1 [file ijms-24-10062-s001.zip › ijms-2323043-supplementary.pdf]

Supplemental Figures

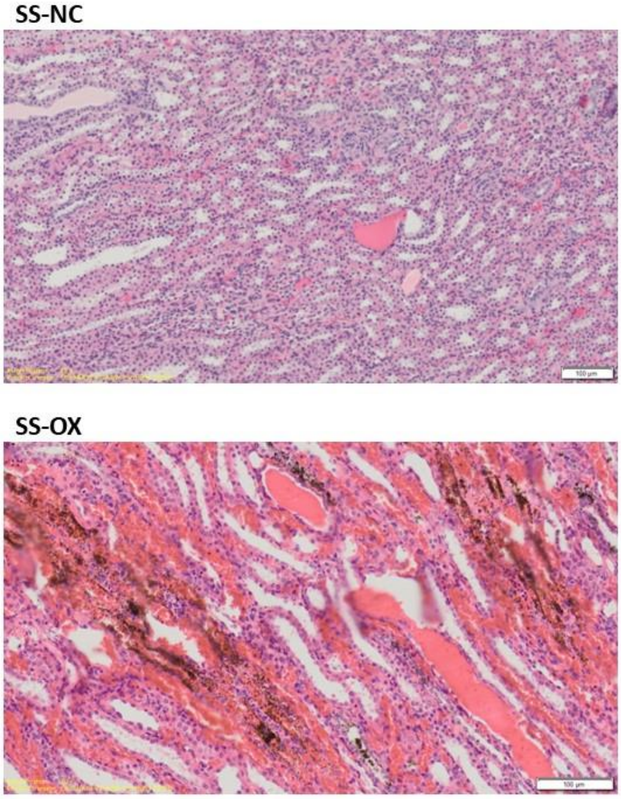

Figure S1. Tubulointerstitial oxalate crystal deposition. Dahl-Salt-Sensitive rats on an oxalate diet (SS-OX) displayed increased calcium oxalate crystal deposition in the renal tubules when compared to Dahl-Salt-Sensitive rats on a normal chow diet (SS-NC)
